# Food culture as a mechanism of social bonding and social identity in primates

**DOI:** 10.3389/fnhum.2026.1788917

**Published:** 2026-04-13

**Authors:** Anna Ilona Roberts, Sam G. B. Roberts

**Affiliations:** 1 Bonobo Foundation, Chester, United Kingdom; 2 School of Psychology, Liverpool John Moores University, Liverpool, United Kingdom

**Keywords:** cultural evolution, cultural transmission, dietary traditions, food culture, primate sociality, social bonding, social complexity, social identity

## Abstract

Food culture is one of the identifying features of social life of any human being every day. The shared habits, rituals and beliefs around producing, procuring and consuming a wide variety of food types, textures and flavours shape how we feel and behave towards others. Food culture defines who we are, our identity and everyday values, and shapes social relationships. This helps us live in complex societies, where we form connections not only with family, but also with society at large and even far-away countries. However, food-related behaviours rarely leave traces in the fossil record, making the evolutionary origins of food culture difficult to reconstruct. Studies of non-human primates help clarify its evolution in the human lineage. Yet research on primate culture has focused largely on social learning and tool use, with relatively little attention given to the cultural dimensions of feeding behaviour. Here we propose that food culture may function as a mechanism of social bonding and social identity in primates, as it does in human groups. Drawing on the Social Brain Hypothesis, we suggest that shared dietary traditions—socially transmitted food preferences—may maintain cohesion in socially complex systems characterised by large groups, fission–fusion dynamics, and tolerant intergroup encounters. Behavioural similarity arising from shared food preferences may facilitate social bonding in complex social systems, providing an additional mechanism when tracking individual relationships becomes cognitively demanding. In humans, cultural behaviours such as food preferences are used to identify others as having the same identity or a different identity. This sense of social identity then affects how we treat others, with members displaying same cultural characteristics favoured over members where these characteristics are absent. This paper proposes that food culture may play a comparable role in primate social systems. We develop a conceptual framework to examine whether dietary traditions are present among primates, contribute to social complexity, influence tactical ranging decisions, and extend beyond feeding preferences to include traditions in vocalisations during feeding. This framework provides testable predictions for understanding how food culture may act as a socio-cognitive mechanism underpinning social bonding and the evolution of human food practices.

## 1 Introduction

One of the key features distinguishing humans from other animals is the scale and cumulative complexity of culture—behavioural expressions that cannot be fully explained by relatedness or local environment and that are shared within social groups (Van Schaik et al., 2012). Indeed, humans transmit a broad spectrum of rich cultural behaviours in the form of social norms, language, customs, buildings, clothes and, of course, food from generation to generation (Samuni et al., 2020). However, as culture does not leave a direct trace in the fossil record, it is unclear when and how culture evolved in our human ancestors (hominins).

In seeking to infer the evolution of culture in humans, a primary focus has been to understand culture in non-human primates (hereafter primates). These studies have widely used the ethnographic method, whereby culture is identified by ruling out possible ecological or genetic explanations for inter-group behavioural variation, thereby inferring social transmission as the most parsimonious explanation. One key example of this method was deployed in the large-scale study of cross-population variation of six chimpanzee (*Pan troglodytes*) communities in 39 candidate behavioural traits (Whiten et al., 1999). This study revealed culture by identifying the presence of habitual or customary behaviours that could not be explained by ecological or genetic differences between populations. Whilst previous research showed culture in behaviours such as technology and communication, foraging behaviours in primates have been omitted because ecological optimums could exist that make culture redundant (Whiten et al., 1999; Felton et al., 2009).

Foraging is one of the most widespread and evolutionarily fundamental behaviours, observed in almost all living animals and, with exception of the few most primitive species, is characterised by complex decision-making processes (Rozin, 2007). Nutritional needs are consistent within primate species (Schoeninger et al., 2015; Raubenheimer et al., 2023; Takahashi et al., 2021; Cui et al., 2025; Izar et al., 2022; Uwimbabazi et al., 2021; Gesquiere et al., 2008), however, there are significant differences in the types of food consumed across populations (Raubenheimer et al., 2023; Cui et al., 2025). In addition to genetic factors (e.g., sensitivity to sweet or bitter taste) and nutritional quality and availability of plants and animals, which constrain food intake, variation in diets may also be influenced by cultural processes (Raubenheimer et al., 2023; Cui et al., 2025; McGrew, 1983; McGrew et al., 1979). McGrew (1983) argues that food culture is a product of social evolution, whereby some foods are selected and some rejected not only as a consequence of adaptation to the ecological environment, but also because of its role in sociality. Of primary interest is capacity to innovate food preferences, and transmit them within the group from generation to generation by some form of social learning of dietary traditions. Social transmission gives rise to behavioural expressions that are usually interpreted in the same or similar way by members of the same culture (Van Schaik et al., 2012). The concept of food culture includes three components: dietary traditions (group specific preferences for frequency and type of food eaten) (Chapman and Fedigan, 1990), modes of behaviour related to acquisition and satisfying food preferences of a given community (e.g., ranging for food; including, but not limited to, tool use in food acquisition) (Luncz et al., 2018; Fuh et al., 2022), and the vocal traditions (e.g., group specific acoustic structure and usage patterns of communication when eating preferred food) (Watson et al., 2015).

Within primates, dietary traditions are well established and include feeding on plant food, which is variable both in frequency and type of species eaten across sites (McGrew, 1983). Comparing plant eating habits across two long-term field sites of chimpanzees in Mahale and Gombe, Nishida et al. (1983) found that among the 143 potential plant food species, only 59% of species have been recorded to be eaten in both areas despite their common occurrence in both sites. Further, group level differences in prey consumption have been claimed among neighbouring primate communities overlapping in space use. Examining prey preferences, Samuni et al. (2020) showed that two groups of bonobos (*Pan paniscus*) differed in their preference for hunting duiker and anomalure (rodent) species, and these behavioural differences were not explained by spatial usage nor by prey abundance, suggesting socially mediated dietary choices. These findings indicate that primates do not select foods solely in response to ecological constraints, but may make dietary choices shaped by social factors, thereby creating a potential pathway through which food culture could intersect with social relationships.

The purpose of this paper is to explore how dietary traditions—socially transmitted food preferences—may function as a mechanism for social bonding and the formation of social identity in primates. Specifically, we examine whether the evolution of cognitive skills underpinning food preferences in primates may have presented a key innovation that facilitated the emergence of more complex, bonded social systems. To evaluate this, we develop a framework in which Parts 3–6 each address a distinct aspect of this hypothesis: Part 3 reviews evidence for the presence of dietary traditions across primate species; Part 4 considers whether dietary traditions contribute to maintaining social complexity; Part 5 explores whether primates make tactical ranging decisions to satisfy socially transmitted food preferences; and Part 6 examines whether food-associated vocalisations reflect culturally transmitted feeding traditions. Together, these sections test the proposition that food culture constitutes a socio-cognitive mechanism supporting complex sociality in primates.

## 2 Conceptual foundations: food culture and social complexity

Primate social relationships—and the social groups that emerge from them—are described as especially complex, and primates have unusually large brains for their body size compared to other mammals (Barton and Dunbar, 1997). The “Social Brain Hypothesis” proposes that forming and maintaining social relationships is cognitively demanding, driving the evolution of large brains (Dunbar, 1998). Primates do not maintain equally strong social relationships with all group members, but form stable, long-lasting bonds with both related and unrelated group members. One of the primary mechanisms that primates use for maintaining these social bonds is grooming, which can account for up to 20% of their total daytime activity budget. The amount of time primates spend grooming is positively related to group size, demonstrating that when groups are large, primates have to spend more time maintaining their social relationships than in small groups (Lehmann et al., 2007). However, the amount of time primates can devote to social activity is limited, because of the demands of other essential activities, notably feeding, resting and moving (Dunbar, 1988).

Further, in human societies, culture is important in social bonding because it signals which social group one belongs to and fosters pro-social behaviour towards this group, in the absence of prior relationships or genetic relatedness (Van Schaik et al., 2012). Social complexity is defined as the network of interactions among many unrelated individuals across different contexts, while cultural complexity refers to the number of distinct, group-specific behaviours. Socially complex societies thus possess more culturally complex traits that differentiate it from other groups (Chick, 1997), and the overlap of culture may facilitate cohesion among groups as a system of interdependent, complementary parts (Chick, 1997). While both similarities and differences in cultural traits shape human social complexity, the contribution of culture—particularly food-related culture—to the social complexity of non-human primates has received little attention (Rozin, 2007).

To bond effectively, primates must predict others’ goals, intentions, and future behaviours (Premack and Woodruff, 1978), which depend on context (e.g., grooming, resting), group membership (e.g., in-group or out-group), relational ties (e.g., kin, allies, rivals), motivations, and demographics. Doing so requires tracking both their own relationships and those among third parties, remembering past interactions, and recognizing individual group members (Kudo and Dunbar, 2001). Empirical studies show that primates use social knowledge of kinship, dominance, and friendships to guide behaviour (Dunbar, 1998); for example, Tonkean macaques respond more strongly to simulated conflicts between friends than non-friends, demonstrating sensitivity to others’ social relationships (Whitehouse and Meunier, 2020).

Animals interpret events in their social environment with regard to their implications for their social relationships. Animals store information about their close social partners preferentially to information about weak or distant social relationships. This is caused by the expectation of further interactions, because information storage is essential for continuing relationship in the future. This information is concerned with individual goals and intentions, stored on animal-by-animal basis. Thus, information about strong social bonds may be stored at the level of individual partners rather than as general social categories (Seyfarth and Cheney, 2003). How individuals evaluate another on the basis of their goals and intentions is equivalent with categorisation of self within social context (Fiske, 2004). Social bonds create a pattern in cognitive processing by including another in one’s own identity (Abrams and Hogg, 1990). The cognitive merging of self with particular others indicates the special role of close social relationships in guiding selective attention that allows emergence of social cohesion (Fiske, 2004). The capacity for social identification (forming cognitive representation of self and others as a part of a larger social unit) is the key feature of large brains and social intelligence in humans that regulates cohesion between individuals within context of their social environment (Fiske, 2004).

In primates, there is a strong positive relationship between brain size and group size, suggesting that the cognitive demands of maintaining stable social bonds have played a key role in primate encephalization (Dunbar, 1998). Group size is constrained both by the time required to maintain social relationships and by the cognitive capacity to process information about these relationships, which sets an upper limit on the number of social relationships that primates can keep track of (Dunbar, 1998). As the number of individuals in a group increases, the number of dyadic and triadic relationships that must be managed grows rapidly, making social complexity inherently higher in larger groups. In smaller groups, primates are able to maintain relationships with all members, whereas in larger groups some individuals become less familiar and social ties tend to weaken, leading to differentiation in relationship strength and limiting the scalability of grooming behaviour (Hill et al., 2008). While this demands that primates in larger social groups manage more complex social relationships, systematic comparisons of how group size influences individual relationships and overall social structure remain limited. Furthermore, the relationship between group size and social structure may depend on the species’ social system.

One key source of variation among primate social systems lies in the temporal and spatial stability of group size and composition. In fission–fusion systems, the broader social group (often termed the *community*) has relatively stable membership. Community size may range from around 20 to more than 100 individuals in species such as chimpanzees and bonobos. However, the internal structure of the community changes dynamically as individuals split into and merge from smaller temporary subgroups (often called *parties*). The size, composition, and duration of these parties vary according to activity (e.g., feeding or resting) and the spatial distribution of resources. As a result, the entire community is rarely observed together in one place, and individuals may remain separated from some community members for extended periods, sometimes weeks. Despite this separation, individuals are able to recognise other community members and maintain long-term social relationships with them. Within primates, the fission-fusion social system is assumed to be more complex than stable groups, because animals rely on social bonding for survival, but the outcome of social relationships is uncertain as frequent spatio-temporal separation makes tracking of social relationships cognitively challenging. This is particularly the case, where association within fission-fusion groups is extended to association with conspecifics between the groups, as is the case in species such as bonobos (Pisor and Surbeck, 2019).

Examining the link between social and cultural complexity of primates living in complex social systems —characterised by large group sizes, fission–fusion organisation, and interactions with neighbouring groups—offers an important opportunity to examine how the underlying social structure affects patterns of association and cultural behaviour. The large group size will result in primates having to keep track of more indirect relationships with whom interaction may be infrequent, as compared to primates that live in smaller groups. Further, keeping track of indirect social relationships with members of fission-fusion communities will impose higher cognitive complexity when compared to species where social groups have low degree of fission-fusion dynamics. Finally, peaceful interactions with members of other communities are cognitively complex, when compared to species where social interactions with neighbouring communities tend to be aggressive. How animals adjust their social strategies and cultural behaviour in complex societies is thus informative of the key cognitive and time-budget pressures involved in sociality in both primates and our human ancestors (hominins).

One such potential strategy is food culture: feeding preferences independent of relatedness or local environment that are shared by members of a social group or between the groups (Van Schaik et al., 2012). Food culture may emerge when the cohesion of complex groups is threatened, placing pressure on groups to adopt costly food preferences (Nettle and Dunbar, 1997). Most studies of primate cultural complexity, in the wild or in captivity, focus on presence or absence of cultural traits. These studies show that there is variation in cultural traits within same groups, which are either habitual (shown repeatedly by several individuals) or customary (shown by most members of at least one age-sex class) (Whiten et al., 1999). However, to develop a real understanding of cultural complexity in primates, several measures of cultural complexity are needed. As well frequency of food types in the diet that can be explained by culture and not nutritional quality or quantity, primates may interact with others through dietary similarity (the degree of overlap) in cultural food types between dyad partners. Thus, individuals with a higher level of cultural complexity may have a greater number of foods in the diet that can be explained by cultural tradition. Further, these culturally complex individuals may have a greater overlap in dietary traditions with group members.

The extent to which diets can act as a social bonding mechanism may be affected by the degree of overlap in the group-specific dietary repertoires between social partners. Dietary similarity between social partners may reflect cognitive processes of social identification, where individuals categorize themselves and others as part of a larger social unit (Byrne and Bates, 2010; van Leeuwen et al., 2020; McGrew and Tutin, 1978; McGrew et al., 2001; Van Leeuwen et al., 2012; Prieur et al., 2024; Badihi et al., 2023; Watts, 2016; Malherbe et al., 2025). Evidence from primates and humans indicates that such social identification regulates patterns of interaction within and between the groups (Baumeister and Leary, 2017; Báez-Mendoza et al., 2021). For instance, a series of studies has shown that dietary similarity is one of the fundamental categories by which humans categorize others into groups. Rozin (2007) shows that food practices act as strong markers of social identity across cultures. When strangers interacted, eating similar food evoked more trust in a cooperation game than when eating dissimilar food items (Woolley and Fishbach, 2017). Further food preferences are formed through long-term social exposure from a young age to food types, textures and flavors preferentially consumed by family and friends and are difficult to modify. These food preferences may therefore have been an important marker of social identity through the course of human evolution, facilitating social bonding across increasingly large and dispersed social groups.

Consistent with this idea, Brito et al. (2023) demonstrate experimentally that individuals who eat or drink together report stronger feelings of communal connection. As communal sharing relationships are expressed through commensality, eating together affirms shared identity within a community (Fiske, 2004). From this perspective, signaling social identity through dietary similarity serves as a particularly strong determinant of social bonds compared with other incidental similarity because shared identities that arise through joint feeding preferences determine another’s goals and intentions as similar to one’s own. In this sense, dietary similarity identifies unrelated strangers as having the same goals and intentions as one’s own on the basis of what one eats (Stajcic, 2013). These shared goals and intentions create feeling of safety, because they are formed in normative contexts that conform to joint identity and prevent individuals from being harmed or exploited. Therefore individuals with a greater cultural complexity in diet have positive fitness outcomes, which arise as a consequence of a stronger sense of joint identity, security and belonging. Dietary preferences do not leave a clear trace in the fossil record, so examining how dietary traditions in primates relate to social bonding can provide insights into the importance of dietary similarity in facilitating both dyadic interactions and large-scale sociality through the course of hominin evolution.

Further, valuable foods provide a key way of exploring how dietary traditions build up to produce the social structure observed at the group level. Valuable foods are those foods that offer a refinement in terms of texture, taste or fat content as well as a distinction because of their quantity or quality (Leroy and Praet, 2015). Primate foods vary in both quality and quantity with meat and fruit being more valuable than foliage because of their higher acquisition cost (Rogers et al., 1990). Ethnographic research revealed that social consumption of large quantities of valuable staple foods (e.g., fruit) maps onto group identities that are more trusting of strangers, whereby individuals forge friendships they can depend on for support. In contrast, valuable foods of very high quality (e.g., meat) are characteristic of forms of social ranking that create or enhance exclusivity (der Van Veen, 2003). While valuable foods can involve higher acquisition effort in terms of locating, processing, or competing for limited resources, in the context of socially transmitted dietary traditions, foods may also be “costly” because they are learned and maintained through social processes. That is, the effort to acquire certain foods reflects not only ecological constraints but also the social and cultural investment required to adhere to group-specific dietary norms. Such costs may function as an honest signal of group membership, because individuals who consistently acquire and consume culturally preferred foods demonstrate commitment to shared social norms.

Taken together, these arguments allow us to derive testable hypotheses about the proximate mechanisms underlying dietary traditions. Specifically, if food culture supports social bonding and identity, then we should expect foraging and food-related behaviour to reflect not only ecological constraints but also socially transmitted preferences within groups. In addition to determining dietary traditions of primates, it is also important to explore the proximate mechanisms behind food selection. The decisions regarding food selection may act purely to satisfy nutritional needs in generalist species whereby foraging decisions reflect simple measures of foods’ nutritional quality and quantity and thus do not involve complex cognition. In contrast, food culture is cognitively complex because foraging decisions are employed flexibly in relation to dietary traditions beyond what would be expected from nutritional considerations alone, which implies that food selection of primates is mediated by social factors.

**Figure 1:**
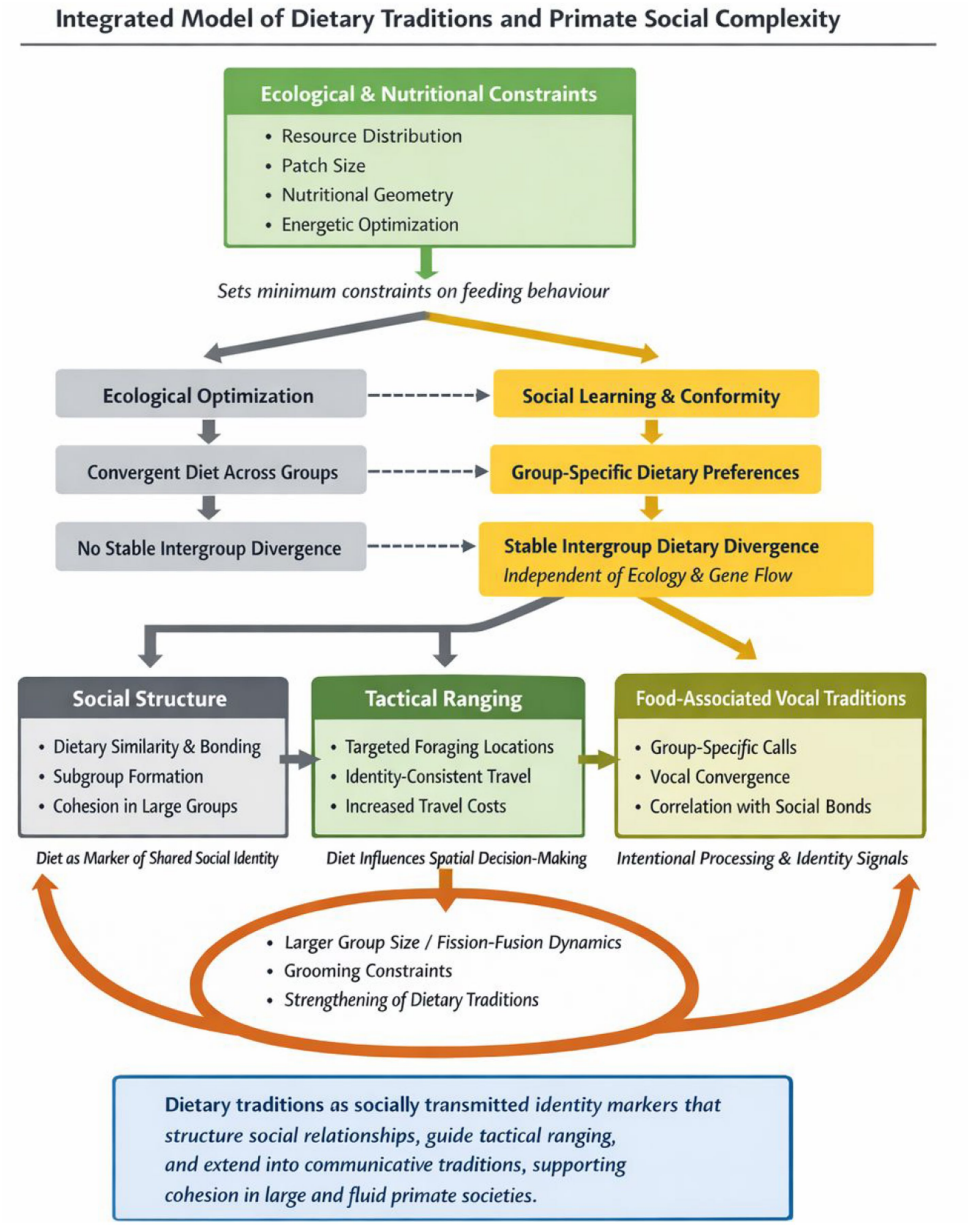
The figure presents a multilevel framework linking ecological constraints, socially transmitted dietary traditions, and their consequences for social systems. Ecological and nutritional factors (e.g., resource distribution, patch structure, nutritional geometry) establish baseline constraints on feeding behaviour. From this foundation, two alternative pathways are depicted: (i) ecological optimisation, in which diets converge across groups in similar environments, and (ii) social transmission, in which learned dietary preferences generate stable differences between groups independent of ecological conditions. These dietary traditions are proposed to have three downstream consequences. First, dietary similarity structures social relationships, contributing to bonding, subgroup formation, and cohesion in large or fluid social systems. Second, culturally valued foods influence ranging behaviour, leading to repeated use of specific foraging locations. Third, dietary traditions extend into food-associated vocalisations, which may function as markers of shared social identity. Feedback loops indicate that increasing group size, fission–fusion dynamics, and tolerant intergroup encounters constrain grooming-based bonding, thereby strengthening reliance on shared dietary and communicative traditions. Together, the model proposes that dietary traditions act as socially transmitted identity markers that structure relationships, guide spatial behaviour, and support cohesion in complex primate societies.

Finally, food culture might be evident in vocal traditions, whereby primates use vocalisations in contexts of feeding on preferred food items for social bonding (Slocombe et al., 2010). Many vocalisations are involuntary reactions to the signaller’s internal emotional state and thus do not involve complex cognition (Cheney and Seyfarth, 2018). In contrast, dietary vocal traditions might be cognitively complex because acoustic structure of communication is mediated by social factors that aligns with group-specific food preferences. Together, dietary and vocal traditions provide a framework for understanding how food culture contributes to the maintenance of complex social systems in primates. An integrated model linking dietary traditions, social bonding, and social identity in primates is presented in Figure 1. This framework illustrates the hypothesized mechanisms by which food culture contributes to the maintenance of complex social systems, which we examine in Sections 3–6. The empirical evidence supporting these four pillars and their associated hypotheses is summarized in Table 1.

**Table 1:** Evidence supporting the four pillars of the dietary traditions model.

Pillar/Section	Hypothesis (H1)	Key empirical evidence	Species/Example	Notes
3. Dietary Traditions	Group-level food preferences are socially transmitted, not solely ecological	Stable intergroup differences in plant and prey consumption; immigrant vervet monkeys adopt host-group diets; chimpanzee crop use differences among neighbouring communities	Bonobos, chimpanzees, vervet monkeys	Dietary divergence persists despite overlapping home ranges and gene flow; magnitude of consumption not fully explained by nutritional quality and availability of plants and animals.
4. Social Complexity	Dietary traditions contribute to maintaining social bonds and group cohesion	Food sharing preferentially occurs among reciprocating partners and allies (bonobos, chimpanzees, golden-lion tamarins), including oxytocin-mediated bonding in chimpanzees. At larger scales, shared attraction to preferred foods influences intergroup encounters, party composition, and tolerant interactions within and between groups.	Bonobos, chimpanzees, golden-lion tamarins	Current evidence links valuable or shareable foods to social effects, but whether socially learned dietary traditions promote social bonding remains unknown because no studies have directly tested this.
5. Tactical Ranging	Primate movement patterns are influenced by socially transmitted dietary preferences	Chimpanzees show stable or differing ranging behaviour despite similar environments, persistent intergroup differences in crop exploitation;	Chimpanzees	Evidence for movement patterns focuses on energetic optimization; preliminary evidence for influence of cultural food value on ranging
6. Food-Associated Vocal Traditions	Vocalizations during feeding reflect group-specific dietary culture and support social bonding	Immigrants’ food-grunt calls converge to match resident group; acoustic convergence occurs after social integration; divergence between neighbouring groups	Chimpanzees	Vocal flexibility independent of nutritional value; socially mediated convergence strengthens social identity; strongest experimental evidence among pillars

## 3 Do primates have dietary traditions?

The primate order, our closest relatives, comprises over 500 species [ITIS lists 528 (Gomon, 2002; Estrada et al., 2017) reports 504] spanning a broad taxonomic and ecological range. Primates display remarkable trophic diversity, reflecting evolutionary adaptation to a wide array of ecological niches. These trophic guilds include insectivores (e.g., *Tarsius* spp.), which consume primarily arthropods; frugivores (e.g., *Ateles* spp.), whose diets consist mostly of fruits and seeds; folivores (e.g., *Alouatta* spp.), which specialize in leaf consumption; folivore-frugivores (e.g., *Colobus* spp.), with a balanced intake of leaves and fruits; omnivores (e.g., *Papio* spp.), which consume both plant and animal matter; and gummivores (e.g., *Callithrix* spp.), which feed predominantly on plant exudates (Galán-Acedo et al., 2019; Hawes and Peres, 2014). This rich ecological and dietary diversity within the primate order, provides a critical comparative framework for exploring the evolutionary origins of food culture.

While some primates specialize in specific foods—such as folivores (leaves) or frugivores (fruit)—many primate species are generalists that consume a broad, flexible array of food types (fruits, leaves, insects, mammals, gums) (Raubenheimer et al., 2023). For instance, bonobos have a broad diet and consume a wide range of animal species such as flying squirrels, monkeys and duikers, which supply essential nutrients (Sakamaki et al., 2016). In addition, plants are an important part of the diet of bonobos (Hohmann and Fruth, 2003) and consist of more than a hundred different species sourced in forests relatively rich in the number of food species they contain, e.g., the plants bonobos eat are 63% of all species in the primary forest, 51% of all species in the secondary forest, and 58% of all species in the swamp (Idani and Asato, 1994). This extensive dietary breadth may arise simply because diverse items are available within their habitat, and broad selection may reflect physiological regulation of nutritional need (Hou et al., 2021).

Traditionally, dietary diversity has been interpreted through optimal foraging theory (OFT), which proposes that natural selection favours strategies that maximize net energetic returns relative to the costs of acquiring food (Felton et al., 2009). Individuals are therefore expected to select prey types, feeding sites, and tactics that yield the highest energy payoff per unit time or effort (Emlen, 1966). Nutritional Geometry theory further emphasizes multidimensional regulation of nutrient intake, showing that animals balance protein, fat, and carbohydrates (Raubenheimer et al., 2023; Takahashi et al., 2021; Cui et al., 2025; Izar et al., 2022) to maintain fitness despite variation in food supply (Whiten, 2021). Field studies of free-ranging primates support this prediction. If occurrence of high quality food sources within the habitat decreases, primates switch to alternative wild foods of lower quality to satisfy nutritional needs (Raubenheimer et al., 2023; Fuh et al., 2022; Hou et al., 2021; Serckx et al., 2014; Pennec et al., 2020). For example, Peruvian spider monkeys (*Ateles chamek*) preferentially consumed figs, but when figs were unavailable, they combined alternative foods in proportions that matched the macronutrient ratios of their preferred diet, consistent with a target intake for protein and non-protein energy (AP: NPE) (Felton et al., 2009). Similarly, baboons (*Papio* spp.) and golden snub-nosed monkeys (*Rhinopithecus roxellana*) maintained consistent AP: NPE ratios over 30–32 consecutive days, despite consuming different food combinations each day (Hou et al., 2021; Johnson et al., 2013).

Other species show similar patterns: mountain gorillas *(Gorilla beringei beringei)* in Virunga and Bwindi forests maintained comparable fiber and macronutrient balances despite seasonal variation in available foods (Rothman et al., 2007; Raubenheimer et al., 2015), and Angola colobus monkeys (*Colobus angolensis*) and black howler monkeys (*Alouatta caraya*) regulated diets to maintain consistent AP: NPE ratios across days and seasons (Dunham and Rodriguez-Saona, 2018; Martínez-Mota et al., 2016). Seasonal and habitat variation in diademed sifaka lemurs (*Propithecus diadema*) and black-and-white ruffed lemurs (Var*ecia variegata*) also showed tight maintenance of macronutrient balance, demonstrating active nutrient regulation (Irwin et al., 2015; Beeby et al., 2023).

Across primate species, a common pattern is protein prioritization, where protein intake is regulated more tightly than non-protein energy. This has been documented in spider monkeys, black howler monkeys, Kenyan blue monkeys, capuchins, golden snub-nosed monkeys, orangutans (*Pongo* spp.), lemurs, and chimpanzees (Takahashi et al., 2021; Izar et al., 2022; Uwimbabazi et al., 2021; Hou et al., 2021; Felton et al., 2009; Johnson et al., 2013; Amato and Garber, 2014; Vogel et al., 2015; Dröscher et al., 2016). Exceptions exist: mountain gorillas maintain constant non-protein energy, rhesus macaques (*Macaca mulatta*) regulate overall energy intake across diets with widely varying AP: NPE ratios, and sifaka lemurs reduce total intake when fat and carbohydrate availability is low to preserve AP: NPE balance (Irwin et al., 2015). These field observations provide strong empirical support for Nutritional Geometry Theory, demonstrating that primates maintain consistent nutrient intake across variable diets, reflecting innate physiological regulation of dietary diversity rather than indiscriminate feeding behaviour (Raubenheimer et al., 2023; Cui et al., 2025; Irwin et al., 2015).

From these ecological perspectives, variation in primate diets is expected to reflect differences in nutritional quality, quantity, distribution, and handling costs of food, rather than socially transmitted traditions. However, many primate diets appear too complex to be fully explained by efficiency-based models alone (Milton, 1979; Post, 1984). The key question, therefore, is not whether primates eat a wide range of foods, but whether the specific composition and relative frequency of foods consumed within particular communities can be fully explained by ecological and nutritional variables, or whether stable, group-level differences remain after such factors are accounted for. Addressing this distinction is essential for determining whether dietary variation represents adaptive nutritional balancing or socially transmitted tradition.

Capturing the diversity of both plants and meat species eaten is important for identifying variation in behavioural patterns among populations of primates to rule out ecological or genetic explanations for group-level differences (Pennec et al., 2020; Masi et al., 2012). Studying neighbouring primate groups, where there is a regular genetic flow between groups overlapping in home ranges, allows the potential sources of bias through genetic influences on food preferences (e.g., sweet or bitter taste preferences) to be excluded. If genetic influences can be excluded, dietary differences between primate groups could be explained either in terms of environmental variation in nutritional quality and availability of plants and animals, or as cultural behaviours that arise as a consequence of social differences among groups (dietary traditions) (Nettle and Dunbar, 1997). Identifying such traditions would indicate that primate diets are shaped by social factors, producing stable group-level differences that are not fully constrained by habitat characteristics.

**Figure 2:**
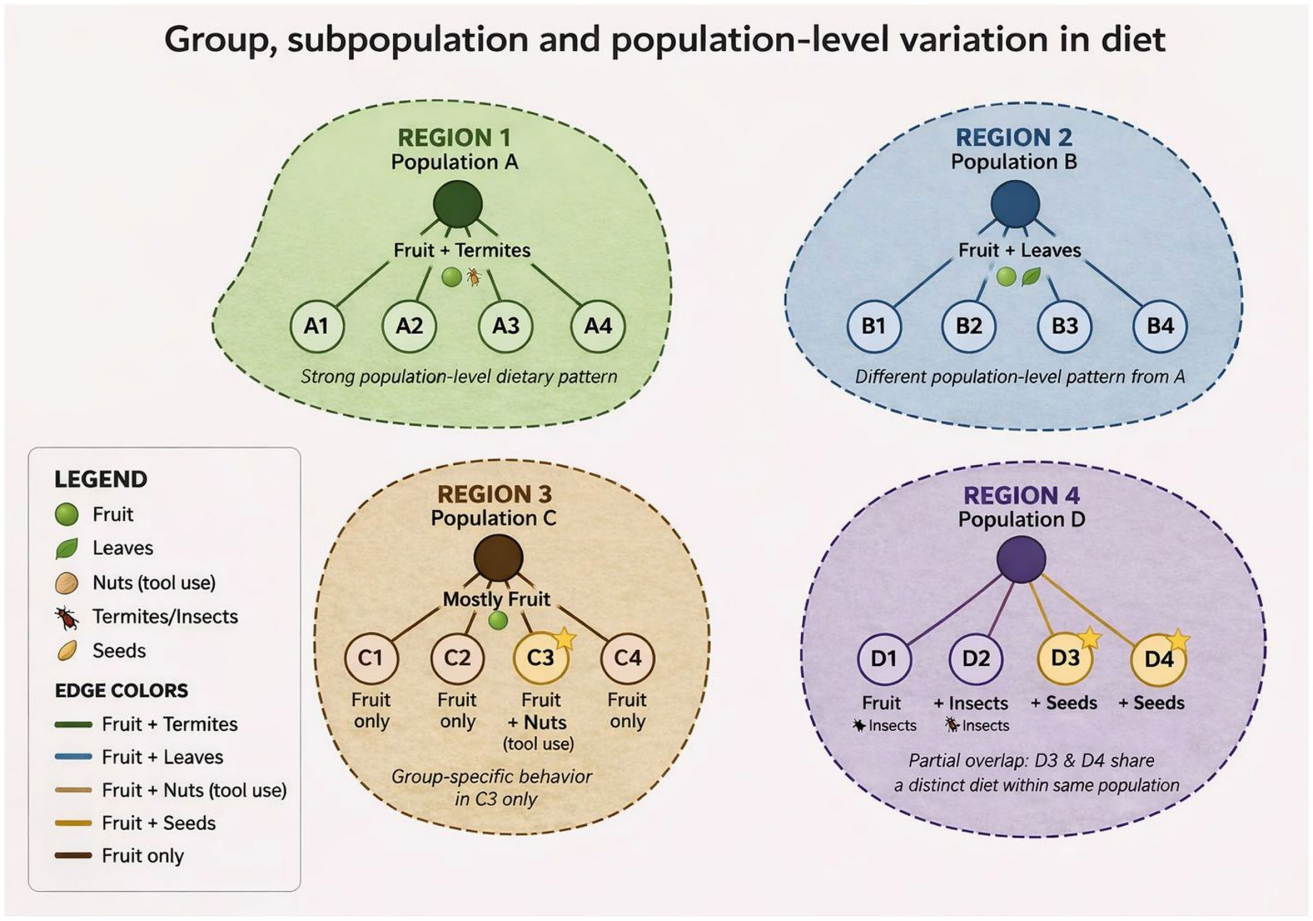
Dietary repertoires emerge through coupled processes of within-group convergence and between-group divergence across social scales. Population-level patterns arise when all groups converge (Populations A–B), subpopulation-level structure when convergence is limited to a subset of groups (Population D), and group-specific repertoires when convergence is restricted to a single group (Population C). Together, these processes generate hierarchical variation in dietary culture.

Several studies provide examples of such complexity that suggest social factors or traditions influence dietary patterns. For instance, larger bonobo groups preferentially hunted duiker and squirrels, whereas smaller groups focused on anomalure, a pattern that persisted even during encounters between groups (Samuni et al., 2020). Immigrant male vervet monkeys (*Chlorocebus pygerythrus*) adopt the foraging habits of their host group (de Van Waal et al., 2013), demonstrating social transmission. Similarly, chimpanzee groups differed in their feeding on human crops, with one group becoming more omnivorous while the other ignored most non-fruit crops (McLennan and Hockings, 2014). These patterns suggest that social factors can shape dietary choices, producing stable, group-specific behaviours that may reflect cultural processes (Pennec et al., 2020; Masi et al., 2012; Nettle and Dunbar, 1997). Although most studies of primate culture examine differences in feeding preferences between groups of the same species, these dietary preferences vary in their scale of expression (See Figure 2 for an integrated overview of the emergence of dietary repertoires). Dietary repertoires emerge through coupled processes of convergence and divergence across social scales. Within-group convergence stabilises shared behaviours among individuals, while between-group divergence differentiates repertoires.

When convergence occurs across all groups within a population, it produces population-level dietary patterns (e.g., Populations A and B). When convergence is limited to a subset of groups, alongside divergence from others, it generates subpopulation-level structure (e.g., Groups D3–D4 versus D1–D2 in Population D). When convergence is confined to a single group, with divergence from other groups, it yields group-specific repertoires (e.g., Group C3 in Population C). Together, these processes produce hierarchical variation in dietary culture, ranging from localised to population-wide expression.

Across these levels, social connectivity shapes dietary similarity by determining opportunities for interaction, while social interaction provides the mechanism through which similarity is established. Because primates typically interact more frequently within than between groups, research has focused on how social structure shapes group-specific feeding patterns. Differences in food consumption may reflect the construction of a ‘social identity’ in primates, whereby individuals with similar diets are perceived as similar and treated as socially aligned by others (Sakamaki et al., 2016). Accordingly, the selection of food species as markers of social identity may lie at the heart of dietary traditions. Identifying these group-specific preferences provides a strong foundation for Studies 4–6, which examine how dietary traditions influence social complexity, guide tactical ranging decisions, and shape food-related communication.

### 3.1 Hypotheses

*H0*: Ecological constraint hypothesis.

Group-level food preferences reflect ecological availability and nutritional optimization.

*Predictions*:

- If a food is present in all groups’ home ranges, all groups will eat it.
- The magnitude of consumption will correspond to the nutritional quality and availability of plants and animals.

*H1*: Dietary traditions hypothesis.

Group-level food preferences reflect socially transmitted traditions independent of ecological constraints.

*Predictions:*

- Foods present in all groups’ home ranges are consumed only by some groups.
- Consumption magnitude does not correspond to nutritional quality and availability of plants and animals.
- Immigrants adopt host-group dietary patterns.
- Divergence persists despite gene flow and ecological overlap.

## 4 What is the role of dietary traditions in maintaining social complexity?

Two main ecological explanations have been proposed to explain variation in primate group size: the optimal group size hypothesis (Terborgh and Janson, 1986) and the ecological constraints hypothesis (Wrangham et al., 1993). The optimal group size hypothesis proposes that groups reach a size at which the benefits of group living outweigh the costs (Rudolph et al., 2019). Benefits include predator defence, access to mates, and collective resource defence. Costs include feeding competition, increased travel, and disease risk. Groups are therefore expected to stabilise near the size that maximises overall fitness. In primates, this pattern has been illustrated in species such as baboons and colobus monkeys, where intermediate-sized groups may experience advantages in predator detection and defence while avoiding the higher feeding competition observed in larger groups (Markham et al., 2015; Snaith and Chapman, 2008).

The ecological constraints hypothesis focuses on limits imposed by food resources (Ekanayake-Weber et al., 2024). Features such as food distribution, abundance, patch size, and renewal rate restrict how many individuals can forage together without intense competition. In chimpanzees, which show fission–fusion dynamics, subgroup size decreases when fruit is scarce or patch size is small (Shibata et al., 2022; Itoh and Nishida, 2007). This pattern reflects limits imposed by food availability. A similar pattern is found in cercopithecine and ateline primates (Van Schaik, 1989; Sterck et al., 1997; Koenig et al., 2013; Emlen and Oring, 1977; Clutton-Brock, 1989). Group fission becomes more likely in large groups that compete over clumped, high-quality, and depletable food patches. Together, these hypotheses show how ecological factors influence both the advantages of certain group sizes and the limits created by feeding competition.

However, ecological factors do not fully explain why group size varies among populations living in similar environments (Rudolph et al., 2019; Henzi et al., 1997). They also do not consistently explain why groups remain stable near these predicted limits. Even when ecological conditions are similar, groups can differ in stability, cohesion, and internal organisation (Shultz and Dunbar, 2022). This pattern suggests that social and cognitive factors also influence group size and complexity. Ecological factors help determine the minimum group size needed for survival and the size that supports efficient feeding (Casari and Tagliapietra, 2018). However, they do not fully explain the maximum group sizes that are observed.

An alternative perspective emphasizes that group size is constrained not only by ecological factors but also by the cognitive and time demands of maintaining social bonds (Dunbar, 1998). As groups grow, individuals face increasing challenges in tracking multiple relationships and managing interactions both within and between groups, including encounters with other communities (Samuni and Surbeck, 2023). Grooming, the primary mechanism for maintaining social bonding, is time-intensive, and as group size increases, grooming effort must be distributed across more partners, reducing the strength and frequency of dyadic interactions (Lehmann et al., 2007), sometimes leading to fission events, as observed in baboon populations (Henzi et al., 1997; Dunbar, 1992). If time and cognitive demands on social bonding become the limiting factor in large and complex social groups, additional bonding mechanisms may be critical in sustaining complex social systems (Aiello and Dunbar, 1993; Nettle and Dunbar, 1997).

Food-related behaviour provides one such potential pathway. Across primates, individuals selectively share food with reciprocating partners and preferred allies, reinforcing existing bonds in species such as bonobos (Fruth and Hohmann, 2018; Yamamoto and Furuichi, 2017), chimpanzees (Wittig et al., 2014; Slocombe and Newton-Fisher, 2005), and golden-lion tamarins (Rapaport, 1997). In chimpanzees, food sharing elevates oxytocin levels in both donors and recipients and is deployed strategically toward socially valued partners (Wittig et al., 2014; Silk et al., 2013), demonstrating that feeding contexts can strengthen affiliative ties. Although food sharing itself reflects reciprocity and relationship quality rather than group-level traditions, these findings show that feeding contexts can serve social functions beyond nutrition.

Beyond individual social bonds, dietary overlap also shapes broader social dynamics: mountain gorilla groups encounter one another more frequently when attracted to the same fruiting trees (Robbins and Sawyer, 2007), and bonobo intergroup encounters are more frequent when abundance of preferred fruit is high (Sakamaki et al., 2018). In both spider monkeys and chimpanzees, the availability and distribution of important and preferred fruits influences the presence of oestrous females in the parties (Matthews et al., 2021; Hartwell et al., 2021). Thus, frugivory has been associated with more tolerant interactions both within and between groups (McLennan and Hockings, 2014; Terborgh and Janson, 1986; Wrangham et al., 1993). Yet most of this evidence concerns the social consequences of valuable or shareable foods rather than socially transmitted dietary traditions (McGrew et al., 1979). As a result, it remains unclear whether dietary traditions per se function as bonding mechanisms highlighting the need for studies that explicitly examine how socially learned food practices contribute to social cohesion and the structuring of primate social systems. If these preferences are culturally maintained and group-specific, they could extend the function of food beyond its nutritional value to operate as markers of shared identity, facilitating social bonding between strangers and unrelated individuals in increasingly complex groups (der Van Veen, 2003; Dunbar, 2017). If dietary traditions contribute to maintaining cohesion in complex social groups, their influence should be detectable in the internal structure of social relationships within those groups. Specifically, overlap between networks based on similarity in dietary traditions and networks based on social interactions may reveal whether dietary similarity plays a role in social bonding. If primates use dietary traditions to structure relationships, individuals who share culturally transmitted food preferences should associate more frequently and form stronger bonds than those with dissimilar diets, independent of ecological overlap.

The internal structuring of primate communities may therefore reflect the complexity of dietary traditions. Subgroups can be defined as sets of individuals who interact more frequently and more strongly with one another than with other members of the wider community (Croft et al., 2010). If dietary traditions function as social bonding mechanism, the presence and composition of such substructures may be associated with similarity in dietary preferences. Likewise, individual differences in social integration may be linked to dietary overlap. Central individuals—those with a larger number of social connections—may share a greater number or diversity of dietary traditions with other group members than more peripheral individuals.

Comparisons of groups that differ in size provide an opportunity to examine whether overall network properties—such as connectedness, density, degree of subgroup formation, and overlap between behavioural networks—covary with the complexity of dietary traditions (Lehmann and Dunbar, 2007; Lehmann and Ross, 2011). If dietary traditions support cohesion in larger groups, we would expect the diversity of such traditions to increase with group size, particularly in fission-fusion social systems.

Finally, peaceful interactions beyond immediate kin and group boundaries are central to the functioning of some primate societies, such as bonobos. If dietary traditions contribute to social complexity, then dietary similarity may also influence intergroup tolerance. Individuals who share culturally transmitted dietary preferences with out-group members may be more likely to engage in peaceful interaction or range overlap (Samuni and Surbeck, 2023). Examining genetically and ecologically homogeneous groups that vary in social structure provides an opportunity to isolate the role of dietary traditions in shaping social complexity. If ecological explanations for variation in social structure can be excluded, then diversity in dietary traditions may account for part of the observed variation in social complexity.

### 4.1 Hypotheses

*H0*: Social complexity reflects ecological and demographic constraints only.

Under this hypothesis, variation in social complexity is determined by ecological variables (e.g., resource distribution, group size, feeding competition) and demographic factors. Dietary overlap between individuals reflects shared exposure to the same environment rather than socially transmitted preferences.

*Predictions*:

- Similarity in diet between individuals will be fully explained by spatial proximity, habitat use, or resource availability.
- After controlling for ecological variables, dietary similarity will not predict patterns of association, grooming, or coalitionary support.
- Measures of social integration (e.g., frequency of association, bond strength, and position within the group’s social network) will be independent of overlap in socially transmitted food preferences.
- Subgroup formation will align with ecological factors such as ranging overlap rather than dietary similarity.
- Variation in group size, fission-fusion dynamics or intergroup tolerance will be explained by ecological and demographic variables rather than by the diversity or overlap of dietary traditions.

*H1*: Social complexity is structured in part by socially transmitted dietary traditions.

Under this hypothesis, dietary traditions function as signals of shared social identity and contribute to the maintenance of cohesion in larger or more fluid groups. Individuals who share culturally transmitted food preferences will preferentially associate, even when ecological factors are controlled.

*Predictions*:

- Overlap in culturally transmitted dietary preferences will positively predict patterns of association and bond strength within groups.
- Individuals sharing similar dietary traditions will be more likely to form cohesive subgroups.
- Measures of social integration will correlate with dietary similarity beyond what is expected from shared habitat use.
- In species characterised by higher social complexity such as large group sizes, fission–fusion organisation, and intergroup tolerance dietary similarity will be especially important in predicting patterns of association.
- Groups with more complex dietary traditions will exhibit larger group sizes, higher fission-fusion dynamics, and intergroup tolerance

## 5 Do primates make tactical ranging decisions to satisfy dietary traditions?

If dietary traditions function as socially transmitted markers of group identity, their influence should extend beyond patterns of consumption to shape spatial decision-making. In this view, food culture would not only determine what primates eat, but also where they travel, how far they move, and which locations they repeatedly target within their home ranges.

The ecological-constraints model posits that living in complex social groups is associated with more complex ranging behaviour which arises as a consequence of higher within-group feeding competition. This is particularly critical for frugivorous mammals, who face periods of scarcity and abundance in the availability of vegetative and reproductive plant parts (Chapman and Chapman, 2000). In rainforest habitats, fruiting trees show a random distribution with complex reproduction patterns, where the timing of fruiting is often unpredictable (Janmaat et al., 2004). Primates must not only track the locations of preferred feeding sites and predict the timing of fruiting, but also allocate movement in ways that maximize energetic returns while minimizing travel costs. Ranging efficiency can have implications for their energy budgets, female fecundity and mortality. Recent studies of constraints on group size suggest that indices of feeding competition can predict a large proportion of the variance in ranging behaviour whenever a larger group travels further per day than a smaller group does to satisfy its food preferences (Fuh et al., 2022). Further, travelling to obtain food sources in primates is not random, but occurs in a goal-directed way as shown by a travel path, whereby a relatively straight-line approach to within the maximum olfactory or visual detection distance of the tree, is followed by a significant change in travel direction to the desired food tree. For example, bonobos seek out ripe fleshy fruits which can be seasonal and patchily distributed in 3–8% of all food trees each month (Inogwabini and Matungila, 2009). In response to the fluctuations in distribution of fruits and seeds, bonobos forage widely in their territories as most of the food trees are scattered throughout the forests at extremely low densities, represented by only one or two trees per species per hectare. Bonobos also hunt prey in large territories of between 35 to 40 km^2^ (Sakamaki et al., 2012), and prey is patchily distributed at 0.7 antelope and 0.3 monkeys per hectare (Wilkie et al., 1998).

Accordingly, travel distances, subgroup cohesion, and home range use are predicted to track spatial and seasonal variation in resource availability. For example, chimpanzees preferentially travel to high-yield fruit trees, and both chimpanzees and spider monkeys (*Ateles* spp.) adjust subgroup size and ranging according to the distribution and density of preferred foods (Janmaat et al., 2013; Chapman et al., 1995; Ban et al., 2016). Seasonal fluctuations further influence movement patterns: vervet monkeys increase travel distances when fruit is scarce (Isbell and Young, 2002), while gorillas, red colobus, and capuchins (*Cebus* spp.) adjust ranging to maintain intake when high-value resources are limited (Fuh et al., 2022; Fragaszy et al., 2004; Trapanese et al., 2019; Inogwabini and Matungila, 2009; Sakamaki et al., 2012; Wilkie et al., 1998; Janmaat et al., 2013; Chapman et al., 1995; Ban et al., 2016; Isbell and Young, 2002; Fragaszy et al., 2004; Trapanese et al., 2019). These findings demonstrate that ecological variables set strong constraints on movement. However, resource availability does not always fully explain ranging patterns. In some populations, movement patterns remain stable despite fluctuations in food abundance, and groups occupying broadly similar environments may display distinct ranging behaviours (Lehmann and Boesch, 2003; Martínez-Íñigo et al., 2021). Such variation suggests that factors beyond immediate energetic or nutritional optimization may influence spatial decision-making.

If dietary traditions are socially transmitted and culturally maintained, they may bias ranging decisions within ecological constraints. That is, among nutritionally viable options, primates may preferentially travel to locations associated with culturally valued foods. Evidence consistent with this possibility comes from neighbouring chimpanzee communities that differ in their exploitation of human crops: one group incorporated a broad range of cultivated foods into its diet, while an adjacent group ignored most non-fruit crops, despite overlapping ranges and access to the same resources (de Van Waal et al., 2013; McLennan and Hockings, 2014). Persistent intergroup differences of this kind suggest that foraging and movement decisions can reflect socially transmitted preferences rather than ecological opportunity alone.

Under this framework, ranging behaviour may serve not only nutritional regulation but also the maintenance of social identity. By repeatedly travelling to and exploiting foods that are culturally important within the group, individuals may reinforce shared dietary traditions and, by extension, group cohesion. Such behaviour would represent a form of tactical ranging in which primates incur additional travel costs to access culturally preferred foods, even when nutritionally comparable alternatives are available at lower energetic cost.

Direct evidence linking movement explicitly to socially learned, group-specific dietary traditions remains limited. Future studies integrating fine-scale movement data, nutritional analysis, and social network measures in neighbouring, genetically and ecologically comparable groups will be necessary to determine whether socially mediated ranging constitutes a strategic mechanism for maintaining food culture.

If ranging behaviour is driven solely by ecological constraints, we would expect goal-directed movement toward food sources to reflect variation in availability, abundance, and nutritional composition. In contrast, if ranging behaviour is shaped in part by socially transmitted dietary traditions, then movement patterns should reflect culturally assigned value to particular foods, even when ecological variables are controlled.

### 5.1 Hypotheses

*H0*: Ecological–ranging hypothesis.

Primate ranging behaviour reflects ecological constraints and nutritional optimization rather than socially transmitted dietary traditions.

*Predictions*:

- Daily travel distances and home range use correspond to the spatial distribution, abundance, and nutritional quality of food resources.
- Ranging patterns change predictably with seasonal fluctuations in resource availability.
- After controlling for ecological variables, groups occupying similar environments exhibit comparable ranging behaviour.
- Movement toward food sources minimizes energetic costs and maximizes intake efficiency.

*H1*: Dietary traditions–ranging hypothesis.

Primate ranging behaviour reflects tactical movement guided by socially transmitted dietary traditions, independent of purely ecological optimization.

*Predictions*:

- Groups with distinct dietary traditions differ in travel routes and home range use despite overlapping ecological environments.
- Individuals incur additional travel costs to access culturally preferred foods, even when nutritionally comparable alternatives are available nearby.
- Immigrant individuals adopt host-group ranging patterns consistent with local dietary traditions.
- Stable intergroup differences in ranging behaviour reflect maintenance of group-specific food culture and social identity rather than ecological opportunity alone.

## 6 Do primates display traditions in affective vocalizations when eating?

Food-associated calls are often described as “functionally referential,” meaning that they may allow receivers to predict events in the environment (Marler et al., 1992). However, an arousal-based perspective suggests that these calls mainly reflect the caller’s excitement in response to finding food, rather than communicating specific information about the food itself (Owren and Rendall, 2001). In this view, receivers respond to the caller’s heightened arousal, not necessarily to a clear signal that specific food is available. Although food calls can sometimes provide additional information—such as food quantity, quality, or divisibility—this information is usually conveyed through changes in call rate rather than distinct acoustic structures (Di Bitetti, 2005). In contrast to alarm calls, which often differ clearly in structure depending on predator type (Blumstein, 1999), most species do not produce unique calls for different foods. When structural differences do occur, they are most often linked to perceived food quality rather than quantity or divisibility (Slocombe and Zuberbuhler, 2006; Clay and Zuberbuhler, 2009). For example, chimpanzees produce acoustically distinct grunt variants corresponding to different food preference classes. Calls to high-preference foods differ reliably in structure and remain stable across trials, suggesting the potential for food-type specificity. However, differentiation is weaker or absent for low- and medium-preference foods, and the evidence does not demonstrate consistent individual labels across all food types. Thus, although rough grunts may carry information about food value, their structure remains closely tied to motivational state. Importantly, the function of food-associated calls varies across species and is closely shaped by socioecology (Slocombe and Zuberbuhler, 2006; Kalan et al., 2015; Leroux et al., 2021; Slocombe and Zuberbuhler, 2005; Chapman and Lefebvre, 1990; Gros-Louis, 2004; Elowson et al., 1991; Roush and Snowdon, 2000). Depending on social structure and ecological pressures, these calls may recruit others to food, reduce predation risk, manage competition, or strengthen social bonds, rather than serve a single unified referential function (Clay et al., 2012).

While food calls are often interpreted primarily through an ecological or motivational lens, recent evidence suggests that their acoustic structure can also be shaped by social processes. In a controlled study of two groups of chimpanzees housed together, immigrant individuals gradually modified the acoustic structure of their food grunts to match those of the resident group following social integration (Watson et al., 2015). Importantly, convergence did not occur immediately upon exposure to the new calls. Instead, acoustic change emerged only after affiliative relationships had formed and subgroup divisions had dissolved. Moreover, call convergence was independent of food preference: individuals’ valuation of the food remained stable across years, while acoustic structure changed significantly.

These findings challenge the assumption that food-associated vocalizations are rigidly coupled to arousal (Clay et al., 2012). Instead, they demonstrate that chimpanzee food grunts are socially flexible and subject to vocal learning processes. The fact that convergence coincided with social integration suggests that call structure may function to signal affiliation or group membership rather than to encode food properties per se. If receivers were already capable of decoding food value prior to convergence, then acoustic similarity is unlikely to have been necessary for referential specificity. A more parsimonious explanation is that vocal convergence served a social function, reinforcing cohesion within the newly integrated community.

In large or fluid social systems, where maintaining bonds is time and cognitively demanding (Lehmann and Dunbar, 2009), vocal convergence in feeding contexts may act as an additional social bonding mechanism. By producing food calls that conform to local acoustic properties, individuals may reduce social distance and increase integration within the network. In this sense, food-associated vocalizations could function as markers of shared social identity. Just as neighbouring communities may differ in dietary traditions, they may also differ in how those foods are vocally expressed. Acoustic similarity would therefore not merely reflect shared arousal, but shared social belonging.

Such socially mediated convergence is particularly relevant in species with fission–fusion dynamics, where subgroup composition changes frequently and maintaining group cohesion requires tracking mechanisms beyond grooming behaviour. In these contexts, food-related vocal traditions may contribute to the maintenance of social identity by aligning communicative behaviour with local acoustic structures. Vocal flexibility in feeding contexts thus provides a plausible pathway through which dietary traditions extend beyond nutritional value of food resources to include socially transmitted communicative patterns.

Beyond vocal learning, food calls may also operate as socially directed signals in a manner consistent with intentional communication. In intentional communication signallers have a goal, and recipients attribute meaning to social interaction based on the identity of the caller, their relationship to the caller, and the interactional context (Roberts et al., 2022; Roberts and Roberts, 2020; Roberts et al., 2012). Intentional signals are typically identified through behavioural markers such as audience checking, response waiting, and persistence (Tomasello et al., 1985). However, communication can also function in an intentional manner from the recipient’s perspective, even in the absence of these overt indicators. In the absence of intentionality indicators such as mutual visual contact—which can signal threat between weakly bonded group members (Damjanovic et al., 2022)—group specific signals may function in a way of intentional signalling (Kampe et al., 2003; Northoff et al., 2006; D'Argembeau et al., 2007; Ames et al., 2008; Hietanen and Hietanen, 2017).

In this view, a vocalization need not display classical production markers of intentionality to influence social cognition. Rather, when acoustic property aligns with group-specific structure, and when convergence occurs in context of social integration, calls may function as intentional signals because they index shared identity (Schäfer et al., 2020; Svensson et al., 2023). Applied to feeding contexts, this perspective suggests that food calls may do more than express arousal or satiety. The signal need not involve explicit response waiting to influence recipients’ social processing. Instead, its group aligned acoustic properties may heighten attention, facilitate cognitive processing of social information at food sites, and facilitate bonded relationships (Roberts and Roberts, 2025; Roberts, 2025).

Under an ecologically driven account, feeding vocalizations would remain tightly coupled to internal states such as excitement or satiety in response to nutritional value of food resources. Under a socially driven account, by contrast, food-associated calls would show preferences in relation to group-specific dietary traditions and social identity of the group. The documented capacity for socially mediated acoustic modification in chimpanzee food grunts supports the plausibility of this latter interpretation, indicating that such calls are not rigid reflexes but communicative acts embedded within social networks.

### 6.1 Hypotheses

*H0*: Ecological-affective hypothesis.

Food-related vocalizations are primarily involuntary, reflecting individual affect or arousal and constrained by ecological factors such as nutritional value of food resource or its abundance.

*Predictions*:

- Acoustic structure and frequency of vocalizations will vary only according to food properties (e.g., size, nutritional value).
- Vocal patterns will be similar across groups consuming identical foods in similar ecological contexts.
- Vocalizations will show limited association with social network structure or social behaviours.

*H1*: Dietary tradition–vocalization hypothesis.

Food-related vocalizations reflect socially transmitted, group-specific traditions that signal social identity, facilitate social bonding, and may include features of intentional communication.

*Predictions:*

- Vocalizations associated with group specific food traditions will differ between neighbouring groups, independent of ecological variation.
- Individuals will adopt the local community’s vocal patterns upon immigration.
- Similarity in feeding vocalizations will correlate with social bonds within the community.
- Food-related vocal traditions will be most pronounced in species or populations with larger group sizes, high fission–fusion dynamics, intergroup tolerance where maintaining cohesion requires supplementary social bonding mechanisms
- Acoustic features and group specific deployment will allow recipients to infer social goals, reflecting intentional communicative processes that enhance social cohesion.

## 7 Discussion

Patterns of culture leave little direct trace in the hominin fossil record and thus to explore the evolutionary origins of human culture, many studies have focused on social learning in animals. Social learning can result in the emergence of subpopulations with characteristic behavioural profiles, creating social barriers, as observed in multiple traditions spanning diverse aspects of animals’ lives, such as tool use, social and sexual behaviours (Whiten, 2021). Culturally mediated population structure has important implications for the survival of the species as it influences species-wide phenotypic diversity and adaptability to changing social conditions (Brakes et al., 2019). It is therefore not surprising that the burgeoning reach of animal culture has seen an explosion in discoveries of culture in a rapidly growing range of animal species, from primates and cetaceans to a diverse range of birds, fish, and also invertebrates (Cantor et al., 2015; Nishida, 1987). This wealth of methodological, empirical and theoretical advances in studies of animal culture provides an important springboard from which to consider deeper questions about evolution of culture in humans.

Previous studies have focused on the social learning mechanisms underlying observed group level differences in behaviour, advancing our progress in understanding the evolution of culture and the cognitive skills underpinning its evolution. In this sense, culture is constructed by forming a mental representation of particular social behaviour, an object or a technique through teaching or imitation of other. For instance, individuals have a true understanding of “kinds” such as tools (e.g., hammers) and representing these as “made for something” (Gruber et al., 2015) or have understanding of types of prey (e.g., duiker) and that it is edible (‘prey image”) (McGrew, 1983; Sakamaki et al., 2016). This approach recognises social evolution as an outcome of genetic drift coupled with dispersion of animal groups across time and space, whereas cultural evolution occurs when imperfect copying of others, combined with group dispersion, leads to behavioural variation and innovation (Whiten, 2021). Characterising culture in a fashion that reflects the process of forming social relationships, whereby individuals acquire food preferences to form a “social identity” is key to our understanding of social complexity but its role in cultural transmission has been omitted. The capacity for social identification (forming cognitive representation of self and others as a part of a larger social unit) is one of the most important features of large brains and social intelligence in humans (Dunbar and Dunbar, 1998). These social identities in human groups are clearly culturally defined and regulated which gives them their character of being unique to each group, rather than present in some groups but not the others. Thus, the current lack of emphasis on social identity in studies of animal culture severely limits the usefulness of culture in understanding *social networks*, where the behavioural similarity is a major component in characterising the interaction between two individuals.

Further, due to a focus on social learning mechanisms, the great majority of culture studies considers only behaviours that are known to occur in one population of animal species but not another, such as ant dipping or tool use for nut-cracking in chimpanzees using a presence/absence approach to identifying cultural variation (Whiten et al., 1999). There is also a tendency to use only networks that characterise complete social networks of individual groups, whilst omitting their connections to other groups. This is an unsatisfactory solution, as cultural diversity is also likely to emerge in extra-community social networks (Pisor and Surbeck, 2019). This hinders the accurate representation of social relationships in species, where social relationships cross intercommunity boundaries. If culture is to fulfil its potential in the study of *social* systems, it is necessary to describe and compare social and cultural complexity across community boundaries, so the nature of the cultural complexity between different groups can be characterised precisely.

To assess the link between social complexity and culture requires a systematic way of defining, measuring and comparing behavioural complexity across groups and species. Currently, there is a lack of such a standardized measure of behavioural complexity and developing such as measure that can be applied across different species has been described as the “grail of social analysis” (Whitehead, 2008). Whilst some behaviours may be evident in only one group or species of animals, feeding behaviour is common to all living animals and, for most species, involves characteristic behaviours. However, almost no study to date has considered the role of feeding preferences as a form of social bonding mechanism that facilitates complex sociality, despite the group level differences in feeding preferences observed in many primate species (Whiten et al., 1999). Thus there is an urgent need to consider feeding data and identify whether feeding interactions play a role in forming and maintaining social relationships (Hinde, 1976). In a complex social system, animals may use cognitively complex feeding preferences, underpinned by forming an image of “social identity” to manage social relationships, whereas in less complex social systems animals may use cognitively less complex feeding preferences that reflect simple measures of nutritional quality and quantity.

Whilst much progress has been made assessing the archaeological record, the study of hominin social life is in its infancy (Dunbar et al., 2014). As hominins are close relatives to non-human primates, and one of the trends in human evolution is of increasing group size over time, understanding how dietary traditions change with increasing group size provides valuable insights into the evolution of human sociality. It is generally well accepted that the split between non-human primates and proto-hominins was marked by a shift in diet composition (Leonard, 2003). This is usually attributed to the complexity of environment, whereby a change occurred in environmental conditions transforming tropical forest into savannah woodlands (Reed and Rector, 2007). As a consequence of changes in climate, hominins were exposed to longer periods of low food availability, which forced them to adopt alternative sources of food such as meat, nuts, or underground storage organs of plants (Tooby and DeVore, 1987). In contrast to models that associate the shift in diets with the changes in specific habitats and food availability, the role of changes in social environments is becoming increasingly recognised. The shift in diet may have been adopted in large social groups, to increase social cohesion by diversifying preferences for costly meat and fruit consumption (Nettle and Dunbar, 1997). Whilst there has been some initial research on influence of group size on cultural diversity in primates (Derex et al., 2013), the work on food culture is still developing. Examining the role of dietary traditions in maintaining primate social complexity provides a new approach with which to examine the archaeological record, with the focus on social factors (individuals or groups) and the behaviour (e.g., dietary traditions). The use of dietary traditions as a social bonding mechanism is just starting to be applied to human evolution (Dunbar, 2017), and a more complete understanding of how food culture and sociality are linked in primates would provide key insights into the role of food culture in human evolution.

## 8 Conclusion

Over the course of human evolution, food-based markers of social identity appear to have played an important role in facilitating social bonding across increasingly large and dispersed social groups. Ethnographic and archaeological evidence suggests that shared food practices—from feasting traditions to status-linked displays—have contributed to the formation and maintenance of stable social relationships and emerging sociopolitical complexity (Mir et al., 2025; Haggis, 2007). These patterns represent culturally transmitted systems of shared meaning and practice surrounding food, and thus provide clear evidence of food culture as a driver of social organisation in human evolution. However, it remains unclear whether the use of such culturally shared food practices to structure social relationships reflects deep evolutionary roots or represents a uniquely derived feature of human societies (Dunbar, 2017).

In this paper, we have advanced a theoretical framework for addressing this question in a comparative and evolutionary context. We propose that food culture—defined as socially transmitted dietary traditions—functions as a mechanism for social bonding and social identity in primates, with important implications for the evolution of complex social cognition. Specifically, we argue that evaluating the role of food culture in primate social complexity requires attention to four interrelated issues: whether primates exhibit dietary traditions; how such traditions contribute to maintaining social relationships within and between groups; whether individuals make tactical ranging decisions to satisfy shared dietary practices; and whether food culture extends beyond feeding preferences to include traditions in vocalisations during feeding. Together, these lines of inquiry provide a foundation for empirically assessing food culture as a socio-cognitive mechanism supporting social cohesion in primates, and for clarifying the evolutionary continuity between primate and human forms of social bonding, identity formation, and cultural signalling.

## Data Availability

The original contributions presented in the study are included in the article/supplementary material, further inquiries can be directed to the corresponding author.
